# A simple formula for enumerating comparisons in trials and network meta-analysis

**DOI:** 10.12688/f1000research.17352.2

**Published:** 2019-04-03

**Authors:** Farhad Shokraneh, Clive E. Adams

**Affiliations:** 1 Cochrane Schizophrenia Group, Division of Psychiatry and Applied Psychology, Institute of Mental Health, School of Medicine, University of Nottingham, Nottingham, NG7 2TU, UK

**Keywords:** Pairwise Comparisons, Study-Based Registers, Clinical Trials, Randomised Controlled Trials, Network Meta-Analysis, Systematic Reviews

## Abstract

We present use of a simple formula to calculate the number of pairwise comparisons of interventions within a single trial or network meta-analyses. We used the data from our previous network meta-analysis to build a study-based register and enumerated the direct pairwise comparisons from the trials therein. We then compared this with the number of comparisons predicted by use of the formula and finally with the reported number of comparisons (indirect or direct) within the network meta-analysis. A total of 133 trials of 8 interventions were selected which included 163 comparisons. The network of these showed 16 unique direct comparisons. The formula predicted an expected 28 indirect or direct comparisons and this is the number that were indeed reported. The formula produces an accurate enumeration of the potential comparisons within a single trial or network meta-analysis. Its use could help transparency of reporting should a shortfall occur between comparisons actually used and the potential total.

## Introduction

The pairwise comparisons reported within each randomized controlled trial are being documented in study-based registers ^1^. This lends itself to accurate indexing and enumeration of these comparisons within the studies and then subsequent supply of immediate, highly sensitive and highly specific search results to those wishing to investigate one or more particular comparisons within systematic reviews and meta-analyses or overviews and network meta-analysis ^1, 2^.

To gain a perspective on the absolute effectiveness of a treatment it is ideal to compare all the existing medications with placebo and for relative effects with each other in pairwise comparison trials. However, some of pairwise comparisons of the medications have not been tested within trials at all. Finally, even if some of the possible pairwise comparisons have been directly tested within trials not all may be eligible for inclusion in a network meta-analysis ^3^. This leaves a gap between the research has been done and the research that should or could have been undertaken and finding this highlights gaps in the fair testing of treatments ^4^.

A two-arm trial will generate one pairwise comparison. A three-arm trial, however. will generate three, and a six-arm study, 15 pairwise comparisons. It is easy to lose track of how many comparisons one study can generate. This is more likely when it comes to the many direct, indirect or mixed comparisons within a network. This paper describes a simple formula for enumerating the possible number of comparisons within a single trial or planned network meta-analysis in advance.

## Methods

### The formula

The formula below solves this where n is the number of arms in a single study or network and N is the number of pairwise comparisons:

$$N=(n^{*}(n-1))/2$$

Where *n* > 0;

*n* is a natural number;

Then every intervention is compared to every other intervention except itself so: n*( *n*-1);

Because *N* is a bidirectional comparison ( *X* vs. *Y* = *Y* vs. *X*) so: ( n*( *n*-1))/2;

This is an established formula from combinatorics for calculating number of pairs for a number of items in a set.

**Figure 1.**
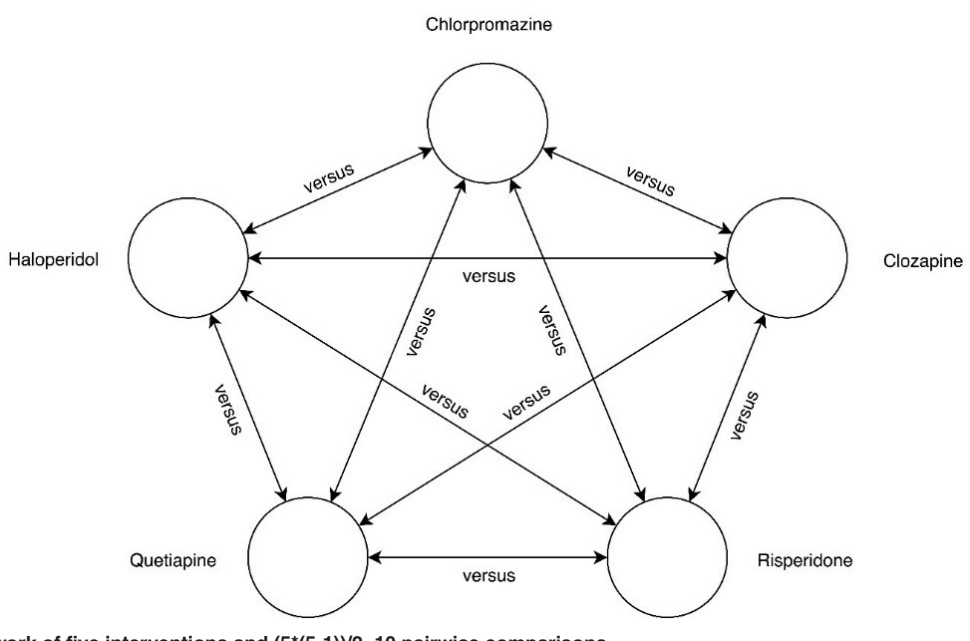
Network of five interventions and (5*(5-1))/2=10 pairwise comparisons.

The networks of 2 to 10 interventions will create networks in shapes of line, triangle, rectangle, pentagon, hexagon, heptagon, octagon, nonagon and decagon, respectively. A visual proof of a network of five interventions and (5*(5-1))/2=10 pairwise comparisons is presented in Figure 1.

Adding any new intervention to the trial or network will create *n*-1 new pairwise comparisons. For example, where there are 6 arms in a trial—or 6 nodes in network meta-analysis—there will be (6*(6-1))/2=15 comparisons; adding a new intervention (6+1=7) will create 7-1=6 new pairwise direct comparisons in an individual trial and 6 direct or indirect comparisons in a network meta-analysis. Although this formula has been used for other purposes such as Metcalfe’s law in telecommunication, its use in the current context is novel.

### Testing the formula: working back from existing network meta-analyses

We used the open data ^5^ from our previously published network meta-analysis ^6^ to re-create and enumerate the comparisons within the network. Using the direct comparisons reported in the trials within the network, we applied the formula and then compared the number of potential or expected comparisons (formula-derived) and the actual or observed number reported within the network analysis.

## Results

### Number of direct and indirect comparisons

**Figure 2.**
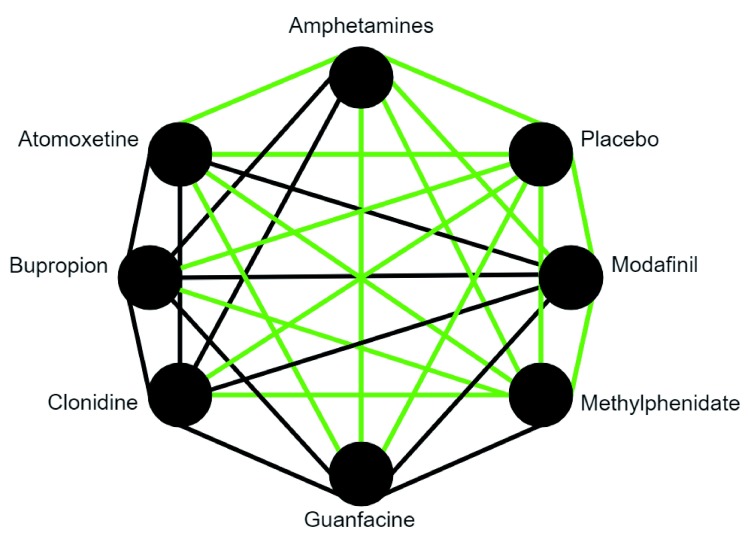
All the possible unique bidirectional comparisons of 8 ADHD medications. Only 16 out of 28 comparisons have been directly compared in trials (green lines).

We built a small study-based register based—thus avoiding the pitfall of multiple counting—containing all 133 included studies in our previous network meta-analysis ^6, 7^. These trials reported comparisons from 8 interventions. Using our formula, 8 interventions should create 28 unique comparisons: (8*(8-1))/2=28 ( Figure 2).

### Reported comparisons within the trials

We extracted the separate intervention arms from the open data to re-create the direct comparisons from within trials. The trials had either two or three arms so each study could create either two or three comparisons. As a result the 133 studies had 163 comparisons, the majority of which were duplicated. After removing these duplicates, this created 16 unique direct comparisons with between 1 and 47 studies per comparison for 8 interventions ( Table 1). These 16 observed comparisons are 57% of the 28 expected by use of the formula above.

**Table 1.** Direct comparisons extracted from trials and their associated studies.

Comparison	Number of studies	Study tag
Amphetamines vs. Atomoxetine	1	Wigal 2005 (SLI381-404, NCT00506727)
Amphetamines vs. Guanfacine	1	Taylor 2001
Amphetamines * vs. Methylphenidate	6	Coghill 2013 (SPD489-325); Efron 1997; Plizka 2000; SPD489-405 (NCT01552915); SPD489-406 (NCT01552902);Stein 2011 (NCT00393042)
Amphetamines vs. Modafinil	1	Taylor 2000
Amphetamines vs. Placebo	21	Adler 2008b (NRP104.303, NCT00334880); Adler 2013 (SPD489-403, NCT01101022); Biederman 2002 (SLI 381-301); Biederman 2007 (NRP104-301, NCT00248092); Biederman 2012 (2008P000971, NCT00801229); Coghill 2013 (SPD489-325); Findling 2011 (SPD 489- 305, NCT00735371); Frick 2017 (SPD465-303, NCT00152022); Kay 2009a; Paterson 1999; Plizka 2000; Spencer 2001; SPD489-405 (NCT01552915); SPD489-406 (NCT01552902);Spencer 2006 (SLI381-314, NCT00507065); Spencer 2008 (SPD465-301, NCT00150579); Stein 2011 (NCT00393042); Taylor 2000; Taylor 2001; Weisler 2006 (SLI381-303); Winhusen 2010 (NCT00253747)
Atomoxetine vs. Guanfacine	1	Hervas 2014 (SPD503-316, NCT01244490, EudraCT: 2010- 018579-12)
Atomoxetine vs. Methylphenidate	8	Bedard 2015 (NCT00183391); Newcorn 2008 (B4Z-MC-LYBI); Sangal 2006 (B4Z-US-LYAV); Schulz 2012; Spencer 2002a (B4Z-MC-HFBD); Spencer 2002b (B4Z-MC-HFBK); Wang 2007 (NCT00486083, B4Z-MC-LYBR (6934)); Weisler 2012 (NCT00880217)
Atomoxetine vs. Placebo	41	Adler 2008a (B4Z-MC-LYBV, NCT00190931); Adler 2009a (B4Z-US-LYDQ, NCT00190879); Adler 2009b (B4Z-US-LYCU. NCT00190736); NCT00190736); Allen 2005 (B4Z-MC-LYAS); Arnold 2006; Bain 2013 (NCT00429091); Bangs 2007 (B4Z-MC-LYAX); Bangs 2008 (B4Z-MC-LYBX, NCT00191698); Block 2009 (B4Z-US-LYCC, NCT00486122); Dell'Agnello 2009; Dittman 2011; Durell 2013 (B4Z-US-LYDZ, NCT00510276); Gau 2007 (B4Z-TW-S010, NCT00485459); Geller 2007 (B4Z-US-LYBP); Goto 2017 (B4ZJE-LYEE, NCT00962104); Harfterkamp 2012 (NCT00380692); Hervas 2014 (SPD503-316, NCT01244490, EudraCT: 2010- 018579-12); Kay 2009b; Kelsey 2004 (B4Z-US-LYBG); Lin 2016 (NCT00917371); Martenyi 2010 (B4Z-MW-LYCZ, NCT00386581); McRae-Clark 2010 (R21DA018221, NCT00360269); Michelson 2001 (B4Z-MC-LYAC); Michelson 2002 (B4Z-MC-LYAT); Michelson 2003a; Michelson 2003b; Montoya 2009 (B4Z-XM-LYDM, NCT00191945); Newcorn 2008 (B4Z-MC-LYBI); Spencer 1998; Spencer 2002a (B4Z-MC-HFBD); Spencer 2002b (B4Z-MC-HFBK); Sutherland 2012 (NCT00174226); Svanborg 2009 (B4Z-SO-LY15, EUCTR2004-003941-42-SE, NCT00191542); Takahashi 2009 (B4Z-JE-LYBC, NCT00191295); Wehmeier 2012 (B4Z-SB-LYDV, NCT00546910); Weisler 2012 (NCT00880217); Weiss 2005 (B4Z-MC-LYAW); Wietecha 2013 (NCT00607919); Wilens 2008 (B4Z-MC-LYBY, NCT00190957); Wilens 2011 (NCT00528697); Young 2011 (B4Z-US-LYCW, NCT00190775)
Bupropion vs. Methylphenidate	2	Jafarinia 2012; Moharari 2012 (IRCT201012295500N1)
Bupropion vs. Placebo	4	Casat 1989; Reimherr 2005; Wilens 2001; Wilens 2005 (NCT00048360)
Clonidine vs. Methylphenidate	4	Connor 2000; Kurlan 2002; Palumbo 2008 (NCT00031395); van der Meere 1999
Clonidine vs. Placebo	5	Jain 2011 (NCT00556959); Kurlan 2002; Palumbo 2008 (NCT00031395); Singer 1995; van der Meere 1999
Guanfacine vs. Placebo	12	Biederman 2008 (SPD503-301, NCT00152009); Connor 2010 (SPD503-307, NCT00367835); Hervas 2014 (SPD503-316, NCT01244490, EudraCT: 2010- 018579-12); Kollins 2011 (SPD503-206, NCT00150592); McCracken 2016; NCT01069523; Newcorn 2013 (SPD503-314, NCT00997984); Rugino 2014 (NCT01156051); Sallee 2009 (SPD503-304, NCT00150618); Schahill 2001 (NCT00004376); Taylor 2001; Wilens 2015 (SPD503-312, EUCTR2011-002221-21, NCT01081132)
Methylphenidate vs. Modafinil	1	Amiri 2008
Methylphenidate vs. Placebo	47	Abikoff 2009; Adler 2009c (CR011560, NCT00326391); Biederman 2006a (subsample of NCT00181571); Biehl 2016; Bron 2014; Buitelaar 1996; Casas 2013 (EudraCT: 2007-002111-82); Childress 2009 (CRIT124E2305, NCT00301236); Coghill 2013 (SPD489-325); Cook 1993; CRIT124DUS02; Dopfner 2003; Findling 2008 (NCT00444574); Ginsberg 2012 (EUCTR2006-002553-80-SE); Goodman 2016 (NCT00937040); Greenhill 2002; Greenhill 2006b (CRIT124E2301); Grizenko 2012; Herring 2012 (NCT00475735); Huss 2014 (CRIT124D2302, EUCTR2010-021533-31-DE, NCT01259492); Kooij 2004; Kurlan 2002; Lin 2014 (NCT00922636); Medori 2008 (LAMDA-I EUCTR2004- 000730-37, NCT00246220); Newcorn 2008 (B4Z-MC-LYBI); Palumbo 2008 (NCT00031395); Philipsen 2015 (EUCTR2006-000222-31-DE, ISRCTN54096201); Plizka 2000; Reimherr 2007; Rosler 2009; Schrantee 2016 (NTR3103, EUCTR2010-023654-37-NL); Simonoff 2013 (ISRCTN683849); SPD489-405 (NCT01552915); SPD489-406 (NCT01552902); Spencer 1995; Spencer 2002a (B4Z-MC-HFBD); Spencer 2002b (B4Z-MC-HFBK); Spencer 2005; Spencer 2007 (CRIT124E2302); Stein 2011 (NCT00393042); Takahashi 2014 (NCT01323192); Taylor 1987; van der Meere 1999; Weisler 2012 (NCT00880217); Wender 2011; Wigal 2004; Wigal 2015 (NCT01239030)
Modafinil vs. Placebo	8	Arnold 2014 (C1538/2027/AD/US, NCT00315276); Biederman 2005 (Study 311 Cephalon); Biederman 2006b; Greenhill 2006a (Study 309 Cephalon); Kahbazi 2009; Rugino 2003; Swanson 2006; Taylor 2000

* Amphetamines include Lisdexamfetamine.

### Direct comparisons eligible for network meta-analysis

**Figure 3.**
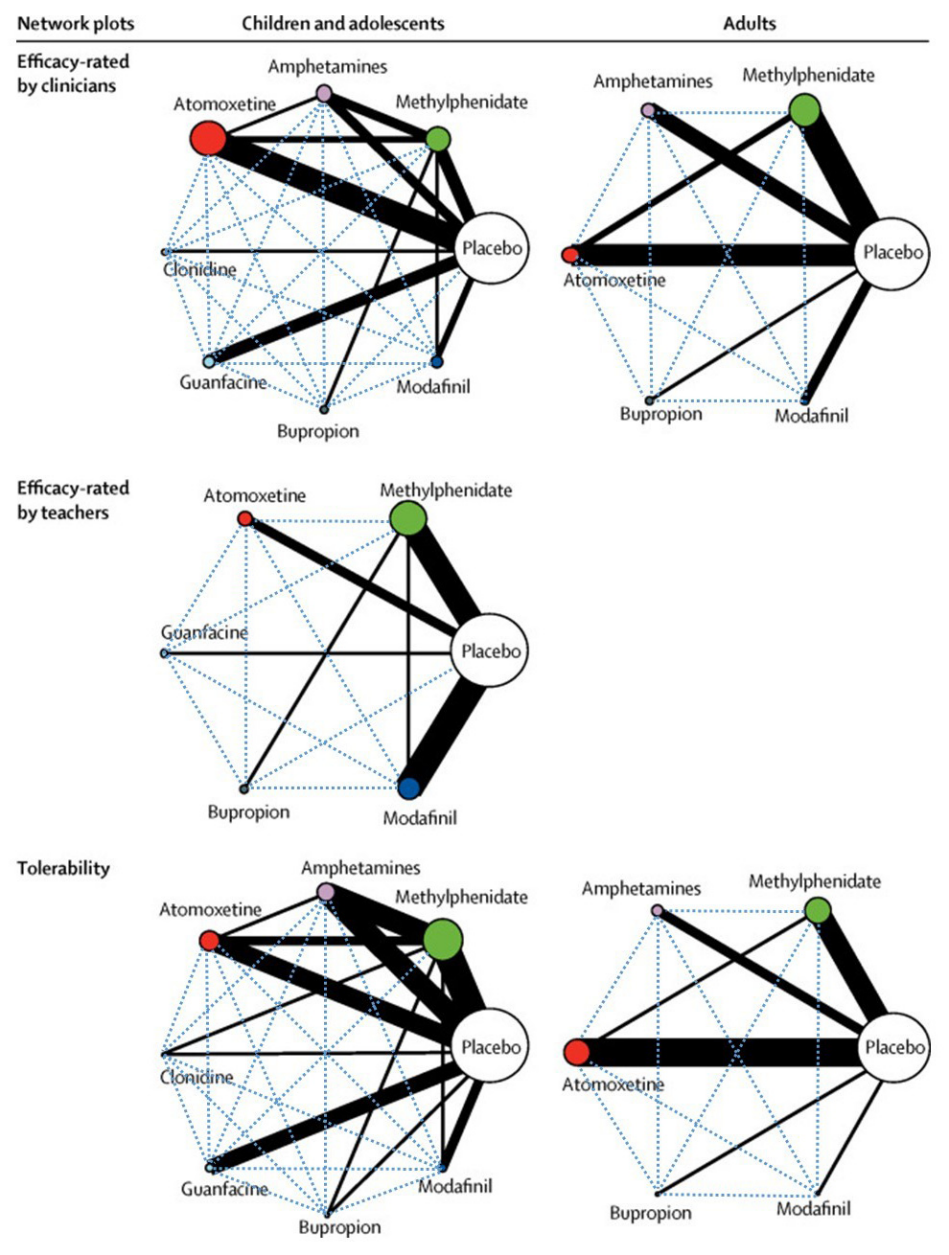
Direct and indirect comparisons in the network meta-analysis of 8 interventions for primary outcome. (Dark lines are eligible comparisons for pairwise meta-analysis, added dotted blue lines show indirect comparisons). This image has been modified from Cortese *et al*. 2018 ^6^ under Creative Commons Attribution License (CC BY).

Among five networks reported in the final paper, the number of comparisons in these five network meta-analyses, however, varies from 6 (for 3 networks) to 11 (for 1 network) and 13 (for 1 network) ( Figure 3). As visualized in Figure 3, only 21.42% to 46.42% of comparisons were eligible for pairwise meta-analysis ( Table 2).

**Table 2.** Comparisons from the body of evidence.

Source of comparisons	Type of comparisons		Eligibility for analyses		# of comparisons	% of comparisons
	Direct	Indirect	Eligible	Ineligible		
Formula	√	√	√	√	**28=**(8*(8-1))/2	100.00
Randomised trials	√	**×**	√	√	**16** ( Table 1)	57.14
Pairwise meta-analysis	√	**×**	√	**×**	**6-13** ( Figure 3) *	21.42 to 46.42
Network meta-analysis	√	√	√	√	**28** ( Figure 2)	100.00

* There are five networks in Figure 3 and each has 6, 11, or 13 eligible comparisons. Three out of 16 comparisons from trials have not been included in any of five network plots.

### Comparisons in network meta-analysis plots

**Figure 4.**
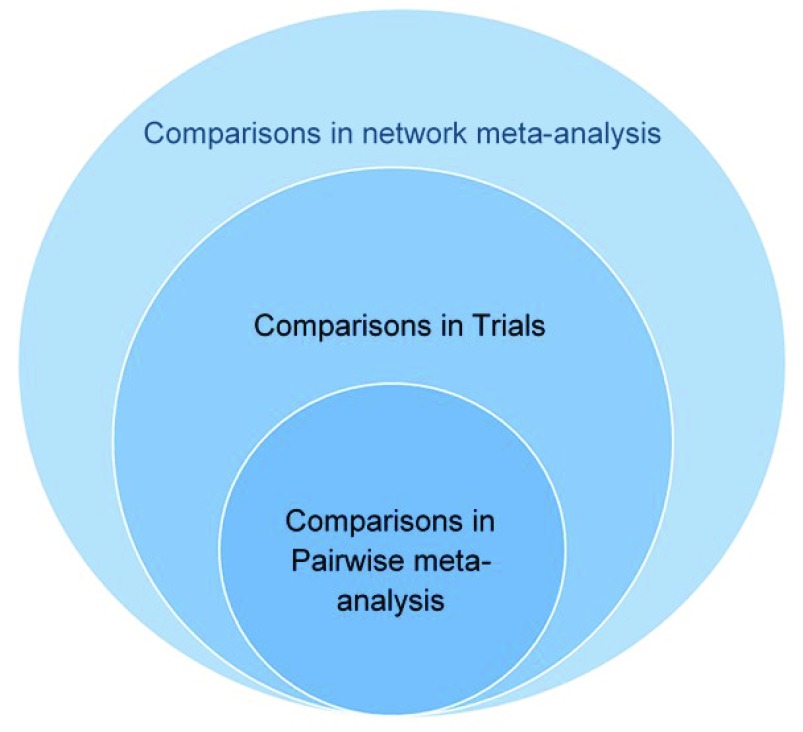
Venn diagram showing the coverage of comparisons by the network meta-analysis (from formula), and pairwise meta-analysis (from network plots), and trials (from study-based register).

From Figure 3 we can calculate that about 42% of comparisons expected through use of the formula have not been tested directly in trials. This is a direct evidence-gap. The number of missing comparisons varies between nine out of 15 in three networks with six interventions, 17 out of 28 in one network with eight interventions, and 15 out of 28 in another network with eight interventions ( Figure 3). However, all 28 comparisons expected by use of the formula were utilized and reported within the network meta-analysis. It is possible that some of the comparisons predicted by the formula would have been deemed ineligible—either by adherence to a network review protocol or through *post hoc* exclusions—but this was not the case in this particular review ( Figure 4). This diagram shows that only some of the comparisons from trials in study-based register could be included in pairwise meta-analysis. In addition, the number of comparisons in network meta-analysis (calculated by formula) is larger and inclusive of all the comparisons in the network of interventions and includes all the possible unique comparisons even if the comparisons are not in trials or in pairwise meta-analysis.

## Discussion

This formula can be employed when estimating the total number of comparisons (direct and indirect combined) theoretically possible within a proposed network meta-analysis. It would be possible that there would sometimes be a discrepancy between the number of comparisons *theoretically* possible and those actually employed within any given network meta-analysis. The formula would highlight this for researchers and readers and, before and after analyses, facilitate descriptions of why particular comparisons have not been included.

## Conclusion

The formula produces an accurate enumeration of the potential comparisons within a single trial or network meta-analysis.

Any shortfall between the full potential of the data and the actual number of comparisons within a network meta-analysis should be possible to explain through reference to pre-stipulated eligibility criteria or *post hoc* exclusions.

## Data availability

The data analyzed in the present study have been published previously ^6, 7^.
