# Supplementary material for: A simple formula for enumerating comparisons in trials and network meta-analysis
Source: F1000Res. 2019 Apr 3;8:38. Originally published 2019 Jan 9. [Version 2] doi: 10.12688/f1000research.17352.2 (PMC6402080; doi:10.12688/f1000research.17352.2)
Supplement: Peer review history

# Peer review: Reviewer response for version 1

**DOI:** 10.5256/f1000research.18976.r44117

**Published:** 2019-03-04

**Authors:** G. Mustafa Soomro

**Affiliations:** 1 St. James Hospital, Portsmouth, UK

This is a useful paper for demonstrating and discussing how an established formula could be used for calculating all possible pairs of comparisons for interventions in a network meta-analysis. Thus, reviewers would be able to find out how many potential comparisons have not been carried out.

 I have some minor suggestions numbered from 1) to 4):

1. In the abstract the following sentence should be amended for clarity perhaps as follows: “A total of 133 trials included in the network generated 163 comparisons (16 unique direct comparisons for 8 interventions)”. Amendment: “A total of 133 trials of 8 interventions were selected which included 163 comparisons. The network of these showed 16 unique direct comparisons.”.
2. On page 2, it says that N is a triangular number. Either this point is not relevant, or why being a triangular number is important should be described.
3. I think they should say that the formula they have used is an established formula from combinatorics for calculating number of pairs for a number of items in a set.
4. On page 3 under “Comparisons in network meta-analysis plots”, line 2 and line 11 “though” should be changed to "through".

I have read this submission. I believe that I have an appropriate level of expertise to confirm that it is of an acceptable scientific standard.

# Response

**Published:** 2019-03-27

**Authors:** Farhad Shokraneh

**Affiliations:** University of Nottingham, UK

Dear Dr G. Mustafa Soomro,

Thanks for spending your time for reviewing our work and commenting on it. In the following lines, we replied to your comments and made changes to the paper to cover your suggestions.

**COMMENT**: This is a useful paper for demonstrating and discussing how an established formula could be used for calculating all possible pairs of comparisons for interventions in a network meta-analysis. Thus, reviewers would be able to find out how many potential comparisons have not been carried out. I have some minor suggestions: In the abstract the following sentence should be amended for clarity perhaps as follows: “A total of 133 trials included in the network generated 163 comparisons (16 unique direct comparisons for 8 interventions)”. Amendment: “A total of 133 trials of 8 interventions were selected which included 163 comparisons. The network of these showed 16 unique direct comparisons.”

**REPLY**: Thank you. We revised the sentence as suggested.

**CHANGE**: “A total of 133 trials of 8 interventions were selected which included 163 comparisons. The network of these showed 16 unique direct comparisons”.

**COMMENT**: On page 2, it says that N is a triangular number. Either this point is not relevant, or why being a triangular number is important should be described.

**REPLY**: We agree. We deleted this part.

**CHANGE**: “N is a triangular number” was deleted.

**COMMENT**: I think they should say that the formula they have used is an established formula from combinatorics for calculating number of pairs for a number of items in a set.

**REPLY**: Thank you for adding this. We agree and we added your suggestion in the text right after formula.

**CHANGE**: “This is an established formula from combinatorics for calculating number of pairs for a number of items in a set”.

**COMMENT**: On page 3 under “Comparisons in network meta-analysis plots”, line 2 and line 11 “though” should be changed to "through".

**REPLY**: Thank you for detecting these errors. We collected both.

**CHANGE**: we replaced ‘though’ with ‘through’.

Thanks again for your valuable comments.

Best Regards,

Farhad Shokraneh

# Peer review: Reviewer response for version 1

**DOI:** 10.5256/f1000research.18976.r43427

**Published:** 2019-02-13

**Authors:** Julie Broderick

**Affiliations:** 1 Department of Medicine, Division of Physiotherapy, Trinity Centre for Health Sciences, Trinity College Dublin, Dublin, Ireland

In my opinion, this is a novel approach which allows the quantification of comparators in trials and network meta-analyses. Following on from this, the comparators which are possible versus how many were reported can be discussed in further depth. This formula has great applicability in terms of network meta-analysis methodology which is still very much a developing area. 

 Some very minor points; 

1. Please add a table which demonstrates the use of the formula to quantify how many comparators are possible for trials from 2 interventions up to 10. 
2. Pg 3, paragraph with the heading 'Reported comparisons within the trials' - line 2 change 'there to 'the'. 
3. Explanation of Figue 4 in text is not clear. 

I have read this submission. I believe that I have an appropriate level of expertise to confirm that it is of an acceptable scientific standard.

# Response

**Published:** 2019-03-27

**Authors:** Farhad Shokraneh

**Affiliations:** University of Nottingham, UK

Dear Dr Julie Broderick,

Thanks for spending your time for reviewing our work and commenting on it. In the following lines, we replied to your comments and made changes to the paper to cover your suggestions.

**COMMENT**: In my opinion, this is a novel approach which allows the quantification of comparators in trials and network meta-analyses. Following on from this, the comparators which are possible versus how many were reported can be discussed in further depth. This formula has great applicability in terms of network meta-analysis methodology which is still very much a developing area. Some very minor points; please add a table which demonstrates the use of the formula to quantify how many comparators are possible for trials from 2 interventions up to 10.

**REPLY**: Thanks for your positive comment. We added a single visual proof for this formula to show how it works. The networks of 2 to 10 interventions will create networks in shapes of line, triangle, rectangle, pentagon, hexagon, heptagon, octagon, and decagon.

**CHANGE**: We added: “The networks of 2 to 10 interventions will create networks in shapes of line, triangle, rectangle, pentagon, hexagon, heptagon, octagon, and decagon”.

**COMMENT**: Pg 3, paragraph with the heading 'Reported comparisons within the trials' - line 2 change 'there' to 'the'.

**REPLY**: Thank you and sorry for this typo.

**CHANGE**: We change ‘there’ to ‘the’.

**COMMENT**: Explanation of Figure 4 in text is not clear. 

**REPLY**: We added a few sentences in the text to clarify it.

**CHANGE**: “This diagram shows that only some of the comparisons from trials in study-based register could be included in pairwise meta-analysis. In addition, the number of comparisons in network meta-analysis (calculated by formula) is larger and inclusive of all the comparisons in the network of interventions and includes all the possible unique comparisons even if the comparisons are not in trials or in pairwise meta-analysis”.

Thanks again for your valuable comments.

Best Regards,

Farhad Shokraneh
